# Comparative Analysis of Phenotypic and Molecular Data on Response to Main Pear Diseases and Pest Attack in a Germplasm Collection

**DOI:** 10.3390/ijms24076239

**Published:** 2023-03-25

**Authors:** Leontina I. Simionca Mărcășan, Ion Oltean, Sergiu Popa, Mariola Plazas, Santiago Vilanova, Pietro Gramazio, Adriana F. Sestras, Jaime Prohens, Radu E. Sestras

**Affiliations:** 1 Department of Horticulture and Landscape, University of Agricultural Sciences and Veterinary Medicine Cluj-Napoca, 3–5 Manastur Street, 400372 Cluj-Napoca, Romania; leontina.marcasan@usamvcluj.ro (L.I.S.M.); rsestras@usamvcluj.ro (R.E.S.); 2 Department of Plant Protection, University of Agricultural Sciences and Veterinary Medicine Cluj-Napoca, 3–5 Manastur Street, 400372 Cluj-Napoca, Romania; ion.oltean@yahoo.com; 3 Department of Horticulture and Forestry, Technical University of Moldova, 42 Mircesti, 2049 Chisinau, Moldova; sergiu.popa@h.utm.md; 4 Institute for the Conservation and Improvement of Valencian Agrodiversity (COMAV), Universitat Politècnica de València, Camino de Vera s/n, 46022 Valencia, Spain; maplaav@btc.upv.es (M.P.); sanvina@upvnet.upv.es (S.V.); piegra@upv.es (P.G.); 5 Department of Forestry, University of Agricultural Sciences and Veterinary Medicine Cluj-Napoca, 3–5 Manastur Street, 400372 Cluj-Napoca, Romania

**Keywords:** breeding, cultivars, fingerprint, fire blight, genetic resources, pear scab, *Pyrus*, psylla, *Septoria*, SSRs

## Abstract

The pear is an important fruit tree in temperate areas, but due to its sensitivity, fruit yield and quality are often affected by disease and pest attacks. Pear genotypes from a germplasm collection comprising 13 *Pyrus* species, 17 Romanian varieties, and 50 non-Romanian varieties from a worldwide assortment were investigated in this study. Throughout four years, response to attack of the principal pathogens and pests was investigated phenotypically under natural conditions of infection and infestation. SSR markers were used to analyze the genetic diversity of the genotypes. A standardized method for the evaluation of responses to biotic stressors was proposed, which highlighted significant differences between genotypes. The species and varieties with the lowest degrees of attack (*DA*%), calculated based on the frequency and intensity of attack, were identified for pear scab (*Venturia pyrina*), septoria (*Septoria pyricola*), fire blight (*Erwinia amylovora*), and psyllids (*Psylla* sp.). These accessions could provide valuable sources of genes of interest to develop resistant varieties in new pear breeding programs. By combining phenotypic and molecular analyses, significant information was obtained that can be exploited to generate high variability for selection through artificial hybridization by harnessing accessions with complementary molecular fingerprints and high genetic distances.

## 1. Introduction

Pears are one of the world’s oldest cultivated fruits; evidence exists that people in the Neolithic period cared for wild trees and tended to their fruit [1,2,3]. Charred whole fruits of wild pear (*Pyrus* sp.) were commonly found in the Balkans, specifically in Serbia [1,2,3]. Pear cultivation began in China around 7000 years ago, and in Europe the ancient Greek poet Homer (8th century BC) described the pear as a ‘gift from the gods’ in his epic poem *The Odyssey* [4,5,6,7]. Pear trees were cultivated and grafted by Roman farmers, and their fruit was highly prized as a valuable commodity along old trading routes throughout the ancient world [8]. Pears have been mentioned in historical records dating back hundreds of years and have been known to grace the dining tables of monarchs in Persia, China, and Rome [9,10]. Over time, as a consequence of the spread of civilization in Asia, Europe, and America, thousands of different varieties were created and eventually spread [11]. 

Pears have a long history in humanity, culture, and the arts, serving as an exquisite still-life subject for artists, dating back to the works of Renaissance Masters. Among the fruit trees, pears represented a pleasant muse for anonymous artists and featured in the representation of fruits in the old popular culture in Europe [7,11]. Consumer preferences, habits, and culture promoted the evolutionary process of selecting and developing pear cultivars, fostering the differentiation between European and Asian pears [12]. From the 16th to the 19th century, a great interest in pear cultivation arose and many new cultivars were developed, especially in France, Italy, and Belgium [13]. In Belgium, Hardenpont (1705–1774) was the first in a long line of breeders to develop valuable varieties of pear that have spread out throughout the world. Among other notable pomologists, Van Mons (1765–1842) stood out as ‘an early apostle of selection’ [14,15] and obtained remarkable results in pear breeding.

Over time, European pears have been bred to be fragrant and smooth-textured, while Asian pears tend to be crisp and milder in flavor [6,16]. Pears are a great source of fiber and a good source of the vitamins C, A, B1, B2, E, folic acid, and niacin when consumed as fresh fruits [17,18]. Usually, they have about only 100 calories per serving and are high in copper, phosphorus, and potassium; have only trace levels of calcium, chlorine, iron, magnesium, salt, and sulfur; and are fat- and sodium-free [17,18]. Pears can be eaten at any meal, although they usually take center stage during dessert. They can be used in puddings, pies, tarts, and cakes, as well as caramelized and brandied [19]. The organoleptic qualities, taste, aroma, composition, and antioxidant properties of pears influence health-conscious customers’ fruit choices [20,21]. Pears are regarded as a hypoallergenic fruit and are high in fiber yet unlikely to have any negative side effects. They are a good choice against a variety of food allergies and various diseases, as well [19,22,23,24].

The wide variety of pear cultivars provides numerous opportunities for orchard culture diversification, the use of rootstocks, and the advancement of culture technologies, as well as for the commercialization of fruits and industrial processing [13,25,26,27]. There are at least 22 recognized species in the genus *Pyrus* [28], with over 5000 pear accessions having been reported worldwide [22]. Nevertheless, it is quite probable that the actual number is significantly higher than this. Hedrick et al. [15] stated in 1921 that more than 3000 cultivars of the European pear (descended from *Pyrus communis* L.) were known, while Teng in 2011 [29] indicated that more than 3000 cultivars originating from *Pyrus ussuriensis* Maxim., *P. pyrifolia* (Burm.f.) Nak., and *P. sinkiangensis* T.T.Yu have been recorded in China. In addition, just like with apples [30], vegetative multiplication and the extension of different clones, heirlooms, and mutations of the same cultivar, each with its own unique local or regional names, synonyms, and homonyms [4,12,31,32], are likely to make the total number of cultivated forms considerably higher than has been thought.

Pear remains a rather vulnerable species to stress factors, despite the fact that the diversity of pear cultivars offers a diversity of responsiveness to abiotic and biotic stressors [12,13,16]. The pear tree is susceptible to damage from a wide variety of pathogens and pests, which affect both yields in orchards and fruit quality [33,34]. Among the many pear diseases, the most dangerous and damaging is fire blight, caused by *Erwinia amylovora*. This bacterium can cause intense attacks as a result of which the crop can be completely compromised [35,36,37]. Fungal diseases caused by pear scab (*Venturia pyrina*) and septoria (*Septoria pyricola*) are relatively common in pear culture in temperate areas, although preventing or fighting them through phytosanitary treatments is easier to achieve compared to fire blight [27,38]. Among the pests, extremely dangerous are psylla species (*Psylla* sp. or *Cacopsylla*), which can cause great production losses and even affect the sustainability of orchards [37,39,40]. These are the most frequent diseases and pests in Romania, including the fruit-growing areas of Transylvania, a historical region inside Romania’s Carpathian arc [37,41,42], as well as in the Republic of Moldova [43,44,45]. They cause significant losses in both fruit yield and fruit quality. As a result, developing new cultivars that are resistant (or at least tolerant) to the attack of these diseases and pests is an important objective of pear breeding. The Horticulture Research Station in Cluj-Napoca, Romania, situated in the country’s northwest, achieved remarkable results in fruit tree breeding, releasing many cultivars [27,46]. However, breeding cultivars with a desired associated response to the attack of different biotic stress agents is extremely difficult in fruit trees [27,30,47].

The success of breeding is closely related to the variability that may be generated and then subjected to selection. In turn, the variability produced by artificial hybridization is determined by the available gene resources and their compatibility with the fruit tree breeding goals [48,49,50]. The more compatible the parents with the desired characteristics that are used, the higher the chance of effective selection [13,30,51]. In this regard, the current study investigated the phenotypic and genetic diversity related to the response of various pear genotypes from a germplasm collection to biotic stress factors frequent in the Carpathian region, including significant diseases and pests. A wide range of genetic material was investigated, including *Pyrus* species, worldwide varieties, and Romanian autochthonous cultivars (old varieties or new breeding creations), with the goal of identifying possible valuable parents for future pear breeding projects.

## 2. Results

### 2.1. Field Evaluation

Among the thirteen *Pyrus* species studied (a group which included two interspecific hybrids, namely, ×*Pyronia veitkii* and ×*Sorbopyrus*), only *P. persica* did not exhibit symptoms of pear scab (*Venturia pyrina*) infection (Table 1). Aside from this genotype, *P. lindlezi*, *P. longipes*, *P. nivalis*, and *P. eleagrifolia* showed an appropriate response to the attack of this fungal disease, as evidenced by a very low degree of pear scab attack (*DA*%). A surprisingly high degree of pear scab attack (*DA*% = 9.4) was recorded in ×*Pyronia veitkii*, an interspecific hybrid between *Pyrus* and *Cydonia* [52].

The amplitude of the degree of attack among the investigated species in response to the attack of septoria (*Septoria pyricola*) was substantially lower than for pear scab. Along with *P. persica* (with no attack), *P. nivalis* and ×*Sorbopyrus* were infested with a slight *DA*% level of the pathogen. The highest *DA*% levels (3.4%) were identified in *P. communis* and *P. malifolia*.

The response to fire blight *(E. amylovora*) infection varied greatly amongst pear species, with an amplitude of *DA*% ranging from 0 to 32.4%. The most affected by the attack were the genotypes: *P. nivalis*, *P. eleagrifolia*, *P. betulaefolia*, *P. cordata*, ×*Sorbopyrus*, and *P. longipes*. The species *P. persica*, *P. malifolia*, *P. lindlezi*, *P. pyraster*, *P. caucasica*, and ×*Pyronia veitkii* were recorded without the occurrence of attack symptoms. It was also noted that *P. communis* was apparently subjected to a slight attack (*DA*% = 0.4).

Within the species of *Pyrus*, the degree of attack recorded for the most dangerous pests of pears (*Psylla* sp.) varied between 0 and 3.9. No psyllids were observed on the leaves of trees from the species *P. lindlezi*, *P. persica*, and ×*Sorbopyrus*. A low infestation was also recorded in other species, which behaved as slightly susceptible to pests under the conditions of the experiment, i.e., *P. nivalis*, *P. pyraster*, *P. malifolia*, *P. longipes*, *P. betulaefolia*, *P. eleagrifolia*, and *P. cordata*. The most susceptible genotypes to psylla attack, both depending on the degree of attack recorded and due to the significant differences between the *Pyrus* species noted for their favorable reactions to psyllid attack, were *P. communis*, ×*Pyronia veitkii*, as well as *P. caucasica*.

Significant differences in response to biotic stress factors (diseases and Psylla infestations) were observed amongst Romanian pear varieties (Table 2). Haydeea had the lowest *DA*% score against pear scab caused by *V. pyrina*, indicating resistance to this fungus, or at least tolerance. After Haydeea, the following varieties displayed the lowest severity of infection with respect to the pear scab disease: Napoca, Ina Estival, Transilvania, Cu miez roşu, Jubileu 50, Adria, and Milenium. The strongest attacks occurred at Cântări, Republica, Virgiliu Hibernal, Roșioară de Cluj, Argessis, and Meda.

The degree of attack for *Septoria pyricola* had a small amplitude among the Romanian varieties, *DA*% being between 0.2 and 2.5%, these extreme values being recorded in two new creations (Primadona and Milenium, respectively) obtained at the Horticulture Research Station of Cluj-Napoca. Besides Primadona, small *DA*% values were registered for Argessis, Haydeea, Jubilee 50, and Republica.

*Erwinia amylovora* strongly affected the cultivars Republica, Cântări, Argessis, Cu miez roşu, Doina, and Roșioară de Cluj. In addition, relatively high values of *DA*% were evident for Virgiliu Hibernal, Transilvania, Milenium, Jubileu 50, and Primadona. However, no fire blight symptoms were noticed during the study years at Napoca, Adria, Haydeea, Ina estival, Meda, and Zaharoasă de vară. Unlike the two fungal diseases (*V. pyrina* and *S. pyricola*), where the amplitude of *DA*% among the varieties was small, regarding the attack of the bacteria that cause fire blight in Rosaceae, the amplitude of *DA*% was large, between 0 and 33.7%, and the reactions of some cultivars contrasting (i.e., from no attack to strong attack).

For psylla attack, among the 17 Romanian pear cultivars, the degree of attack ranged between 0.4 and 5.1%. The Haydeea variety stood out for its adequate response when infested with pests (this being recorded with the lowest *DA*% value). The following varieties were found to have *DA*% values lower than 2.0: Cu miez roşu, Republica, Cântări, and Adria. At the opposite pole, the varieties Roșioară de Cluj, Virgiliu Hibernal, Doina, and Milenium had the highest susceptibility to psyllid infestations. 

Among the 50 international pear cultivars, the degree of pear scab attack oscillated between 0 and 20.3% (Table 3). It was found that the cultivars Er Shi Shinge, Kristalli, Okusankichi, Olivier de Serres, and Précoce Trottier did not exhibit any symptoms of pathogen infection. The mycosis infection rate of *V. pyrina* in the cultivars Curé, Beurré Hardy, Williams Red, Williams Bovey, Butirra Precoce Morettini, Williams, and Plovdivka Parva was likewise low, with *DA*% levels between 0.3 and 1.0. Instead, the cultivars Laxton Superb, Moonglow, Kostliche Von Germen, Triomphe de Jodoigne, Grand Champion, Noiabriscaia, Fondante des Bois, Van Mons, Lincoln, Conference, General Osmanwill, and Madame Ballet were shown to be the most susceptible to the *V. pyrina* pathogen.

The following cultivars had very small *DA*% values (between 0.1 and 0.4%) of *S. pyricola* infection: Williams Bovey, Pierre Corneille, Williams, Imperial, General Osmanwill, Beurré Hardy, Magness, Abate Fetel, Juliusi Selimesi, Moonglow, General Leclerc, and Er Jang Li. Among the most well-known and widespread cultivars that were recorded to have low septoria attack (*DA*% < 1.0) were Olivier de Serres, Seigneur Esperen, Beurré Giffard, Laxton Superb, Triomphe de Vienne, Curé, Pitmaston Duchess, Dr. Jules Guyot, and Beurré Bosc. The highest degree of attack was registered in Kristalli (*DA*% = 7.0), followed by Conference, Grand Champion, Madame Ballet, and Van Mons (*DA*% between 4.7 and 2.7).

Fire blight infection, caused by *E. amylovora* bacteria, showed wide variation among the 50 cultivars, with values of *DA*% between 0 and 40.7%. A total of 15 cultivars, accounting for a proportion of 30% of the foreign types, did not exhibit any symptoms of fire blight. In alphabetical order, they were: Beurré Amanlis, Beurré Bachelier, Beurré du Luçon, Blanquet Precoce, Bristol Cross, Conference, Conseiller de la Cour, General Osmanwill, Grand Champion, Laurence, Magness, Précoce Trottier, Seigneur Esperen, Williams, and Williams Bovey. Small values of *DA*%, between 1.0 and 3.0, were registered for the varieties Beurré Hardy, Imperial, Curé, Beurré Bosc, and Er Shi Shinge. The most susceptible cultivars to *Erwinia amylovora* infection were Bunte Julibirne, Butirra Precoce Morettini, Chang Pa Li, Bonne Louise d’Avranches, Abate Fetel, and Williams Red.

The variation in the degree of attack with *Psylla* sp. was quite large, between 0 and 14.7%. In addition to Olivier de Serres, in which no pests were identified, it is worth noting that Précoce Trottier, Okusankichi, Laxton Superb, Curé, Er Shi Shinge, Triomphe de Jodoigne, Madame Ballet, General Osmanwill, Magness, Moonglow, Chang Pa Li, Pierre Corneille, and Beurré du Luçon had low levels of *DA*%, ranging from 0.1 to 1.0. Instead, Kristalli was the most strongly attacked (*DA*% = 14.7), followed by Butirra Precoce Morettini, Noiabriscaia, Bonne Louise d’Avranches, Beurré Amanlis, Beurré Bachelier, Imperial, Williams Red, and Williams (all with *DA*% ≥ 5.0).

**Figure 1:**
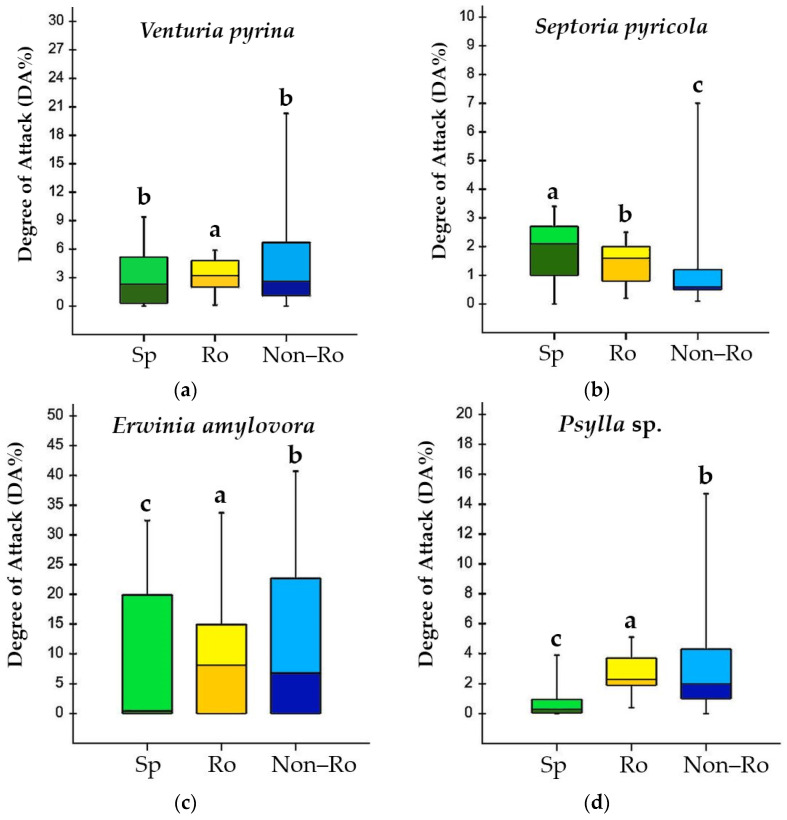
Degree of attack (*DA*%) of the main diseases and pests: (**a**) pear scab (*V. pyrina*); (**b**) septoria (*S. pyricola*); (**c**) fire blight (*E. amylovora*); and (**d**) psylla (*Psylla* sp.). Synthesis of data as boxplots for 13 species (Sp), 17 Romanian cultivars (Ro), and 50 non-Romanian cultivars (Non-Ro). Different letters between groups of genotypes in each boxplot indicate statistically significant differences for the investigated feature at a significance level of *p* < 0.05 (Duncan’s test).

The results regarding the response to the attack of diseases and pests analyzed for the 80 pear genotypes, divided into three groups (species and Romanian and non-Romanian/international varieties), are shown in Figure 1. The boxplots distinguish quite clearly the differences in variation between species and Romanian and non-Romanian cultivars for each examined attribute. Furthermore, the comparisons between groups (using the *DA*% means of the genotypes/groups) reveal significant differences in the responses to the biotic stressors considered in the study, depending on the three genotype categories.

For the response to *V. pyrina* attack, the average values of *DA*% for the *Pyrus* genotypes were the lowest but insignificant compared to the ones recorded for the non-Romanian varieties (Figure 1a). The Romanian varieties appeared to be the most susceptible, with a significantly higher attack than the other two groups but with greater homogeneity in the reaction to pear scab.

The differences between the three groups regarding septoria infection were significant, with the non-Romanian varieties being less susceptible to the disease (Figure 1b). For *E. amylovora*, the boxplots have no bottom whisker, the *DA*% variation is large, and the wild genotypes seem to have the lowest susceptibility (Figure 1c). Significant differences were also identified between the Romanian and non-Romanian varieties, the autochthonous ones having a better tolerance to fire blight. Results like those for fire blight were obtained for the attack of psyllids (Figure 1d). Overall, least susceptible to pests were the *Pyrus* species, followed by the Romanian varieties. The group of cultivars from the international assortment proved to be the most susceptible to attack, but their boxplots reflected high variation in *DA*% levels, as for the other traits.

**Figure 2:**
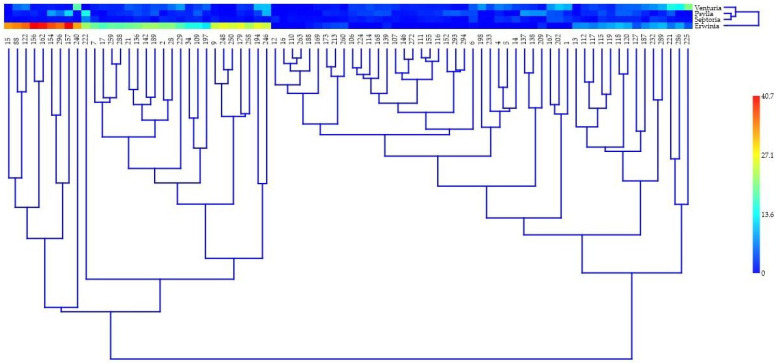
Multivariate analyses performed for 80 pear genotype response as the degree of attack (*DA*%) to three pathogens (pear scab—*V. pyrina*, septoria—*S. pyricola*, and fire blight—*E. amylovora*) and *Psylla* pest assault, using hierarchical clustering, the paired group method (UPGMA—unweighted pair group method with arithmetic mean), and similarity indexes (Euclidean). The genotype codes (numbers) correspond to the names of species and Romanian and non-Romanian cultivars from Table 1, Table 2 and Table 3, i.e., between 1 and 17 are *Pyrus* species, between 21 and 120 are Romanian cultivars, and between 122 and 296 are non-Romanian cultivars from the international assortment.

Multivariate analysis (hierarchical clustering using the UPGMA method and Euclidean similarity indexes) of the *DA*% responses of the 80 pear genotypes to the investigated diseases and pests generated interesting results both for genotype relationships (column dendrogram) and the approach or distance of the analyzed attributes (row dendrogram), along with their heatmap (Figure 2).

The genotype dendrogram is separated into two large clusters, each with numerous sub-clusters and their ramifications. Based on the genotype codes, the small numbers that correspond to the species, as well as the intermediate ones for the Romanian varieties and the large ones for the non-Romanian varieties, are spread and admixed, including up to the level of the smallest subclusters or branches. Thus, there are several small subclusters with very close genotypes for the examined criteria but with no known genetic relationship between them. In an extremely tight subcluster, Haydeea (code 110 (created at the HRS)) is paired with Précoce Trottier (code 263), both of which are paired with *P. persica* (code 16). It is also interesting to note that, among the biotic stress factors, the responses of pear to septoria and psylla appear to be correlated. Their subcluster is coupled above with the response to pear scab, and the response to fire blight attack appears the farthest away.

**Figure 3:**
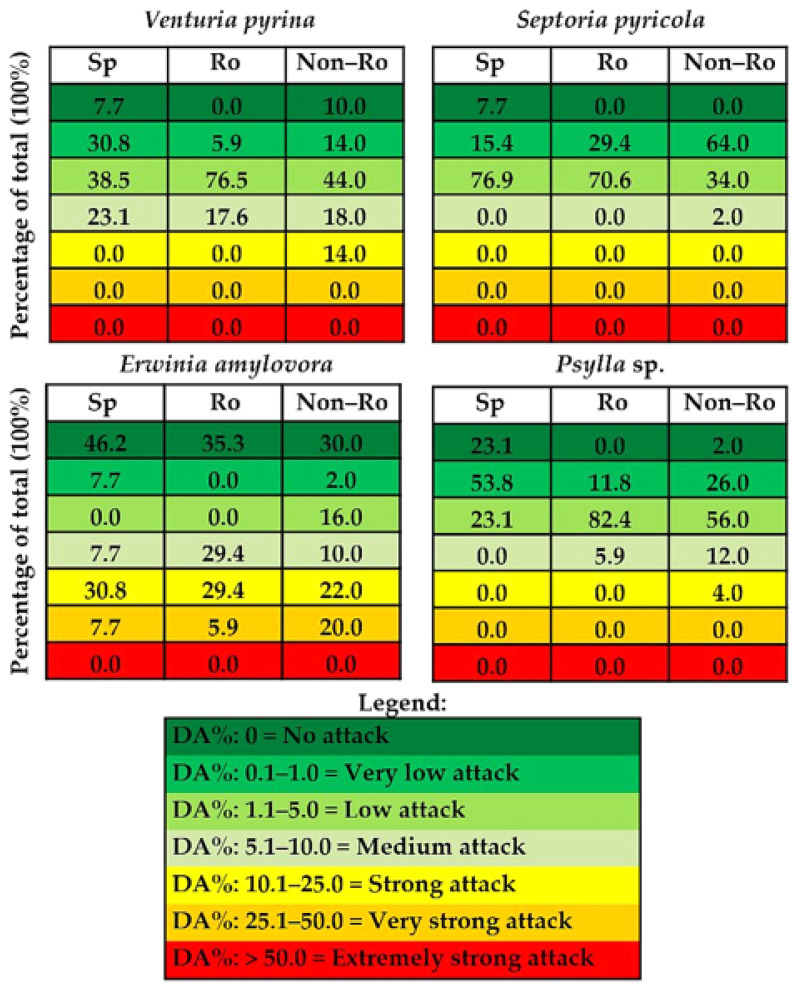
Classification of pear genotypes from the three groups containing 13 species (Sp), 17 Romanian cultivars (Ro), and 50 non-Romanian cultivars (Non-Ro), in attack classes according to the values for degree of attack (*DA*%). Data represent the percentage values of each class from the total genotypes (100%) based on *DA*% of the pear scab (*V. pyrina*), septoria (*S. pyricola*), fire blight (*E. amylovora*), and psylla (*Psylla* sp.).

The classification of pear genotypes from the three groups (species and Romanian and non-Romanian varieties) in seven classes based on the value of the degree of attack (*DA*%), as percentage values of each class from the total of genotypes, reveals that there is no genotype in the class with the highest level of susceptibility, noted as ‘extremely strong attack’ (Figure 3). In addition, in the case of mycotic diseases, there were no genotypes in the ‘very strong attack’ class, and in the ‘strong attack’ class there were 14.0% non-Romanian genotypes for pear scab attack. The largest dispersion of percentages of inclusion in distinct classes, or from a distribution that approaches a certain histogram for a quantitative feature, was reported for *E. amylovora* (even if 50 cases were analyzed for foreign genotypes).

### 2.2. Genetic Analysis

Table 4 presents the genetic diversity statistics of 80 pear genotypes, based on molecular marker analysis of nine SSRs. The major allele frequency was between 0.232 (01D09) and 0.620 (04E03A), with an average major allele frequency of 0.386. A total of 131 alleles were found for the nine SSRs, all of the microsatellites being highly polymorphic. The average alleles per SSR were 14.6, ranging from 4 (04E03A) to 21 (01D09). With a mean value of 0.524, the observed heterozygosity values varied from 0.237 (04E03B) to 0.764 (01F07A). Expected heterozygosity among loci also varied, albeit within narrower bounds, with a mean of 0.755 and values ranging from 0.543 in 04E03A to 0.880 in 01D09. The polymorphism information content (PIC) varied from 0.485 to 0.870, with the lowest value being recorded for 04E03A and the highest for 01D09.

**Figure 4:**
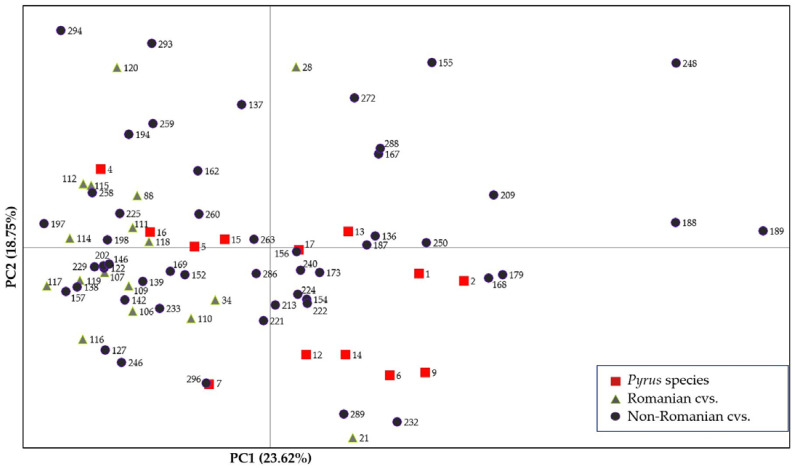
Relationships between the 80 genotypes of pear based on nine SSRs according to the principal coordinate analysis (PCoA). The first principal coordinate is responsible for 23.62% of the molecular variance, the second for 18.75%, and the third for 18.39%. The genotypes were divided into three groups (species, Romanian cultivars, and non-Romanian cultivars), each with its own color. Their codes (numbers) correspond to the names of the genotypes in Table 1 (*Pyrus* species), Table 2 (Romanian cultivars), and Table 3 (non-Romanian cultivars).

Based on the SSR markers, principal coordinate analysis (PCoA) revealed a wide variety of genetic relations between the 80 different genotypes of pear (Figure 4). Within the four quadrants, the greatest data dispersion was found in quadrant I (top right). Instead, the greatest agglomeration appeared in quadrant III (bottom left). Four species (12, 14, 6, and 9; red symbols) stand out relatively close, in quadrant II (bottom left), and a little above them, in the same quadrant, the species with codes 1 and 2. Contrarily, species 4, 15, and 16 are in the opposite quadrant IV. Species 7 is located diagonally with species 13, but in opposite quadrants (III and I), and species 5 and 17 are arranged on the horizontal axis, but on separate sides of the graph. The first principal coordinate (PC1), which explained 23.62% of the genetic variation, separated the species unevenly, eight out of 13 species (1, 2, 6, 9, 12, 13, 14, and 17) exhibiting positive values for PC1, while the rest (4, 5, 7, 15, and 16) exhibited negative PC1 values. The second principal coordinate (PC2), which accounted for 18.75% of the genetic variation, had less discrimination power for the species set, with seven of them (1, 2, 5, 13, 15, 16, and 17) showing values around zero, five showing negative PC2 values (6, 7, 9, 12, and 14), and only one (4) showing a positive PC2 value. Broadly, the species can be divided into three main groups of four accessions. The first group is located in quadrant I, constituted by species 1, 2, 13, and 17; the second group in quadrant II, constituted by species 6, 9, 12, and 14; and the third group in quadrant IV, constituted by species 4, 5, 15, and 16. Finally, the last species (7), plotted alone in quadrant III, is quite distant from the three groups.

Among cultivars, the positive values for PC1 especially discriminate non-Romanian accessions, which are more diverse. Some European varieties of *P. communis* form close groups with other varieties even if they are not necessarily related. Compact groups are formed by the cultivars with codes 107, 122, 146, 202, and 296 from quadrant III; genotypes 154, 222, and 224 (quadrant II); 112, 115 and 258 (quadrant IV); and 167 and 288 (quadrant I). Among them are also new Romanian varieties obtained at the HRS, but which do not have parents in the immediate vicinity or in the groups mentioned, for example, 107—Adria, obtained from artificial hybridization between Napoca and Williams Red; 112—Jubleu 50: Napoca × Butirra Precoce Morettini; and 115—Milenium: Cluj 16–4–12 (Josephine de Malines × Doctor Lucius) × Comtesse de Paris. Roşioară de Cluj (code 118), obtained at the HRS, with very early summer ripening and crimson red fruits, is arranged in a triangle reasonably close to its parents, Williams Red (296) and Beurré Giffard (187). The Romanian varieties are quite compactly arranged, relatively close to the horizontal axis in quadrants III and IV. The exceptions are varieties 21 (Argessis: Napoca × Butirra Precoce Morettini) and 120 (Virgiliu Hibernal: Passe Crassane × Comtesse de Paris), which are distant and in opposition, in quadrants II and IV. In particular, genotype 21 is quite far from all the other Romanian varieties.

Asian accessions 189 (Er Jang Li) and 188 (Er Shi Shinge) are extremely different from accession 197 (General Leclerc) and 157 (Butirra Precoce Morettini), but also from 293 (Williams) and 294 (Williams Bovey). The large distance between the two Asian accessions, as well as the one with code 248 (Okusankichi, which also belongs to *P. pyrifolia*), and the majority of the genotypes emphasizes the significant genetic differences between Asian and European species. It should be noted that close to Williams and Williams Bovey was Virgiliu Hibernal, another variety created by the HRS, and that among the genotypes with the best responses to biotic stressors was Er Shi Shinge. However, the inconsistency in the pear responses, especially to *Erwinia amylovora* attack, depending on the test conditions and the interaction between the genotype and the environment, also emerges from the fact that at NCGR–Corvallis, USA, Er Shi Shinge was cataloged as ‘fire blight susceptible’, as well as Okusankichi (https://www.ars.usda.gov/ARSUserFiles/20721500/catalogs/pyrasian.html, accessed on 6 March 2023).

**Figure 5:**
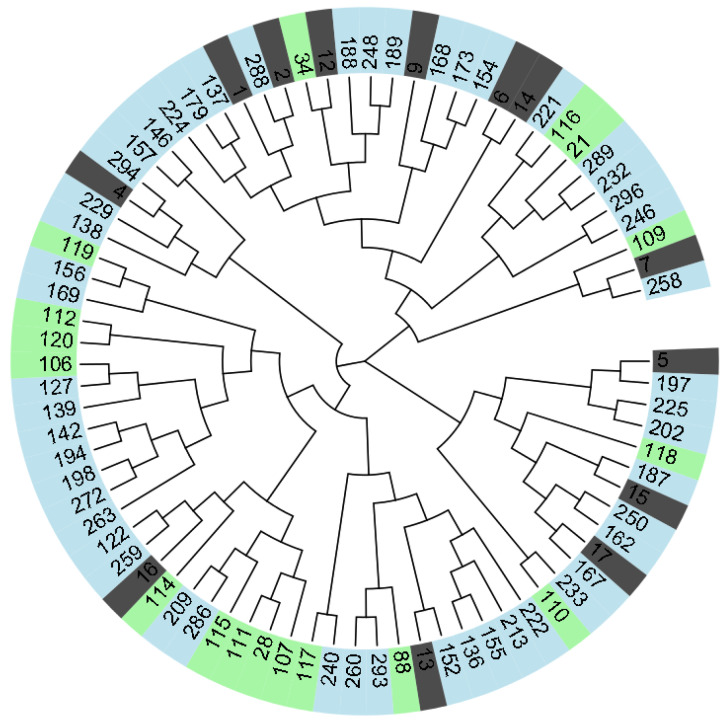
Genetic relationships among 80 pear genotypes revealed by a UPGMA dendrogram, based on nine SSR markers. The dark gray color indicates 13 species of *Pyrus*, the light green color 17 Romanian cultivars, and the light blue color 50 non-Romanian cultivars from the international assortment. Their codes (number) correspond to the names of the genotypes in Table 1 (*Pyrus* species), Table 2 (Romanian cultivars), and Table 3 (non-Romanian cultivars).

The spatial distribution of the genotypes that appear quite randomly in the PCoA for inclusion in the three groups of pears is confirmed by the dendrogram (Figure 5). In this way, three large clusters were differentiated. 

Despite the fact that each cluster has subclusters with various ramifications, they do not entirely or mostly contain only genotypes from one of the three categories suggested in the study. Thus, species and Romanian and non-Romanian cultivars appear quite mixed and placed, sometimes including up to the last ramification level. Genotypes with common colors also appear at the level of small subclusters, including as pairs, reflecting the possible genetic proximity between them.

## 3. Discussion

Improving resistance to diseases and pests remains one of the most important objectives of pear breeding [51,53,54]. Fungal diseases are among the most common pear diseases, affecting both fruit production and fruit quality. Fighting them requires many phytosanitary treatments with fungicides, which can be effective but which have many shortcomings, since they are expensive and increase the cost price of the fruit and have negative influences on the agrobiological and ecological environment and the health of consumers, among other consequences. In addition, they do not always have the expected efficiency, because sometimes climatic conditions, rains, and other different factors can reduce the effectiveness of fungicides [13,28,55]. 

The fungus *V. pyrina* causes pear scab, which is closely linked to the fungus *V. inaequalis*, which causes apple scab. Due to the enormous economic interest, numerous procedures have been developed for the evaluation and monitoring of the main pathogenic agents. In the case of harmful insects, the scenario remains the same [41]. These procedures are of great interest both for orchards, where it is desired to obtain high yields and quality fruits in ecological conditions that are as good as possible for the health of the consumer and the environment, as well as in fruit tree breeding and the development of new cultivars [56]. Over time, the development of methods or procedures for evaluating the response of fruit trees when attacked by diseases and pests that are as objective, correct, and relevant as possible, has been followed. In addition, these methods or procedures should be suitable for species with similar biological and cultural characteristics or for similar pathogens (such as apple scab and pear scab). The test environment, the pathogen isolates, and the natural or artificial conditions in which the infections or infestations occur have a particularly strong impact on the evaluations of resistance or sensitivity to biotic stressors. Therefore, before a phenotypic dataset relating to disease and pest response can be correlated with genetic data, rating scales and context must be assessed thoroughly. Finally, the standardization of data recording and plant phenotypes in response to biotic stressors will contribute to the possibility of correlating these data with genomic data [57]. Postman et al. [57] highlight the importance of standardizing plant disease assessments that could contribute to the identification of resistance genes and their efficient use in fruit breeding. In their work, the main assessment models of disease incidence (GRIN–USDA, IBPGR/IPGRI, etc.) through systems of ratings or scales (i.e., 1 to 5, 1 to 9, etc.), different intervals, their correlations with responses to pathogens (i.e., from ‘very susceptible’ to ‘very resistant’, depending on the scale interval and its ascendent or reverse direction), and the plants or organs on which evaluations are carried out (i.e., foliage or fruit in the case of scab, the portion of the tree blighted in the case of fire blight, etc.) are pragmatically reviewed. These rating systems, rating scales, or phenotypic descriptors for responses to biotic stresses additionally have the benefit of being simple and affordable to use, fast and cheap. As a result, they can be used rather simply in commercial orchards, in germplasm collections with a large number of genotypes, as well as in breeding and testing fields with thousands or tens of thousands of hybrids.

The procedure for the phenotypic evaluation of the responses of genotypes to natural infections with *V. pyrina*, *S. pyricola*, and *E. amylovora*, as well as to natural infestation with *Psylla*, applied in our study was quite laborious. The method used for the determination of the frequency and intensity of attacks, which values were then used to calculate degrees of attack [58,59], was closer to the IBPGR scale adapted by Lateur and Populer [60], which is an assessment key from 1 to 9, than to the evaluation system of the VINQUEST project [61,62], where the grading score is explained by defining the symptoms and the related proportions of affected organs (%).

Therefore, even if the procedures were quite laborious, the recorded data allowed us to apply a reliable statistical calculation using percent of DA instead of ordinal scales in the case of scoring, credit rating scales, or descriptors. There are many studies and debates about disease evaluation terms and concepts and the correctness of the data regarding the assessment of plant diseases [63,64,65], but it is widely agreed that quantitative ordinal disease scales are inherently less accurate since they lack the clarity of a 0 to 100% scale [66,67]. Thus, the genotypes with proper response (which we preferred to call ‘tolerant’, avoiding the term ‘resistant’, which could appear much too subjective) were differentiated quite clearly for each disease analyzed and for the pests represented by psyllids. Additionally, the groups of genotypes included in the three categories (*Pyrus* species, Romanian varieties, and non-Romanian varieties) were statistically differentiated. The results highlighted that the genotypes with the lowest degree of attack (or without attack) to pear scab (*Venturia pyrina*) were: *Pyrus persica*, *P. lindlezi*, and *P. longipes* (among species); Napoca, Ina Estival, and Haydeea (among Romanian varieties); and Er Shi Shinge, Kristalli, Okusankichi, Olivier de Serres, and Précoce Trottier (among non-Romanian varieties). The best responses against septoria were presented by *P. persica* and *P. nivalis* (species); Primadona and Republica (Romanian cvs.); and Williams, Pierre Corneille, and Williams Bovey (non-Romanian cvs.). No symptoms of *E. amylovora* attack were registered in 6 species, 6 Romanian varieties and 15 non-Romanian varieties. The best responses against psylla were registered in *P. lindlezi*, *P. persica*, and *Sorbopyrus* (species); Haydeea and Adria (new Romanian cultivars created at the HRS); and Olivier de Serres, Curé, Laxton Superb, Okusankichi, and Précoce Trottier (non-Romanian cvs.). These genotypes and those with minimal disease and psyllid assault are very likely to include genes of relevance for enhancing resistance to biotic stressors.

The study of the reaction of pear genotypes to stress factors and their use in breeding to create resistant cultivars is of significant interest, and much research is being conducted in this area [13,28,47]. The findings show a wide range of responses, from sensitivity to resistance, depending on the genotype, environment, and culture conditions, pathogens and strains (or biotypes), interactions between various factors, etc. [12,68,69].

Currently, most European pear cultivars (*P. communis*) are considered to be susceptible to *V. pyrina*, which causes pear scab [70]. However, useful genetic resources for improving resistance to this pathogen can be found among them [71,72]. Asian cultivars and some *Pyrus* species also provide a suitable pool of useful genes for disease resistance [68]. By interspecific hybridization between an Asian species and European cultivars, the pear-scab-resistant Euras was created [69]. This is a Romanian cultivar obtained by hybridization ((*P. pyrifolia* × Olivier de Serres) × Doyenné d’Hiver) [73,74]. Variability in response to biotic stressors appears in both cultivars and hybrids produced from parents with different degrees of disease resistance, including pear scab. Due to the wide range of responses to pear scab in F1 hybrids from the cross between Abbè Fétel (Abate Fetel in our study, and registered with *DA*% = 6.7) and Max Red Bartlett (Williams Red in our study, and registered with *DA*% = 1.0), Pierantoni et al. [75] identified quantitative trait loci (QTLs) and categorized seedlings in greenhouse tests as 39% resistant, 33% moderately susceptible, and 28 highly susceptible; major QTLs were found in linkage groups 3 and 7 associated with resistance to pear scab, suggesting two significant genes involved in resistance to *V. pyrina*. Seven QTLs were identified by Won et al. [76] in an interspecific progeny of *P. pyrifolia* and *P. ussuriensis* and were tested against three single-spore isolates of *V. pyrina*. Besides monogenic resistance, which can be found in Asian species but also in European pear cultivars, such as the *Vn* dominant gene which provides significant resistance to pear scab [77], evidence of polygenic resistance has been reported [71,76]. 

While pear scab appears to be more harmful to pear culture than *S. pyricola*, this disease can still have a detrimental impact on crops, their productivity, and their quality. In addition, climatic conditions in some years can contribute to a significant increase in the incidence of septoria disease [78]. This probably explains the differences recorded in the current study compared to a previous one [79] on the same variety (Milenium, created at the HRS, which has a low *DA*% currently but was more sensitive previously), studied in different years and different cultures. Considering the response of trees to the attack of septoria as a quantitative trait and analyzing the general combining ability and the specific combining ability in a half diallel without parents or reciprocal crosses, it was found that both the additive effects of polygenes and the non-additive effects (of dominance and epistasis) contribute to the transmission of sensitivity or resistance to offspring [80]. Since genes of interest related to response to septoria disease can be found in the genotypes noted in the present study, just like in pear scab, linkage maps can identify which markers are connected with the desired trait if resistance is considered to be mediated by several genes [75]. 

Fire blight caused extensive damage in the study region, resulting in the loss of numerous genotypes from the HRS Cluj-Napoca germplasm collection [37]. Previous studies have shown that many widely cultivated cultivars, recognized for their overall value and fruit quality, are highly susceptible to fire blight [81,82,83], and the need for fire-blight-resistant cultivars is considered more pressing than ever [84]. There are various sources of genes identified for resistance to *E. amylovora* [85,86,87], and controlled hybridization is commonly used in breeding programs due to the generally polygenic nature of fire blight resistance and the complexity of its mechanism [88,89,90]. Probably, the complexity of genotypes’ responses and their inconstancy to fire blight attack emerged in the current study from their random classification in attack classes, without the frequency of the distribution resembling a quantitative histogram. The ambiguous responses to bacteria of some genotypes over time, in the same location or close locations, can also be mentioned respecting the current results vs. others previously reported [37,91]. Among the many possible examples, Curé is particularly relevant. This variety has been cultivated for a very long time in Romania and was very popular in old orchards and private gardens in Transylvania. Comparing the data from the present study (where *DA*% = 2.0, considered low attack) with other data from Romania, equivocal results were recorded for Curé, which was rated very sensitive in the south of the country [92] and very strongly attacked in the north of Romania, at a fruit research station in the vicinity of the city of Cluj-Napoca (Bistrița, at about 100 km) [91]. Also to be mentioned is the ambiguity of the response in a previous study at the HRS, where, under the same conditions, in one field trial the *DA*% was 3.0 while in another it was 100.0 (and the trees died) [37]. Another example is Williams (also known as Williams Bon Chrétien, or Bartlett in the US and Canada), the most popular variety of pear in the world, for which a significant quantity of information regarding fire blight response has been published [27]. Although it is usually considered to be susceptible to fire blight [87,93], Williams (but also Williams Bovey) showed no symptoms during the research period, whereas its muted sport Williams Red (Max Red Bartlett), which has an almost identical phenotype, except for the fruit color—red instead of yellow [75]—had one of the highest *DA*% levels recorded, namely, 35.0. The susceptibility of Williams, as well as other cultivars, to fire blight depends on testing, culture, and environmental conditions, either in the field or under controlled conditions, in addition to natural or artificial inoculation, inoculation with different strains, etc. [94,95,96]. In any case, it is widely acknowledged that fire blight is difficult to manage and that a wide range of environmental conditions can influence its development, i.e., disease spread and degree of damage are enhanced by weather, high soil fertility, and ample soil moisture [97,98]. 

The pear sucker species, also known as pear psylla or pear psyllid, are the most dangerous insects in the genus *Psylla* (or *Cacopsylla*, Hemiptera: Psyllidae) [99,100]. They can cause substantial damage to pear tree plantations and have already provoked substantial damage in the HRS area [41,42]. *C. pyri* L. and *C. pyricola* Förster were detected in the collection, with *C. pyri* exhibiting greater dominance in the psylla population. Following a review of the specialized literature, Bell [100] outlined the sources of psylla resistance genes, mainly represented by East Asian species: *P. betulaefolia*, *P. calleryana*, and *P. fauriei*, *P. ussuriensis*, but also some *P.* ×*bretschneideri* hybrids and descendants of *P. ussuriensis* × *P. communis* and *P. pyrifolia* Nakai × *P. communis* and a few genotypes of the European ‘snow pear’ *P. nivalis* Jacq., as well as a few European cultivars belonging to *P. communis* L., with moderate to high levels of resistance (among which are Spina Carpi, an old Italian cultivar, 15 landraces from Eastern Europe, etc.). In previous studies, a high level of resistance to nymphal feeding was noted in ×*Sorbopyrus auricularis* accessions, collected in Romania by van der Zwet et al. [101,102,103]. In our experience, no psyllids were noticed in the nymph stage on the leaves of *P. lindlezi* and *P. persica*. Besides these, the European wild pear, *P. pyraster*, which is considered a subspecies of *P. communis* [27], also responded favorably to both psylla and fire blight attacks.

Even though pathogens and psyllid pressure were lower in the study years than in previous years, when psylla and *E. amylovora* caused significant damage [37,42], including the total loss of some genotypes, the calculation of *DA*% levels confirmed the susceptibility of some valuable and widely used cultivars worldwide. Among the new Romanian cultivars, Haydeea stood out, confirming previous findings that its response to biotic stress factors, including psylla, is appropriate [41].

Molecular investigations revealed intriguing details about the genetic diversity of the pear accessions studied. SSR markers proposed by the European Cooperative Program for Plant Genetic Resources (ECPGR) [104,105] were utilized, and among the 80 genotypes analyzed in our study were some well-known ones, namely, Abate Fetel, Conference, and Williams, recommended for allele determination by Evans et al. [105]. These markers revealed only 4 to 21 alleles per locus, compared to 21–38 in a prior study based on biological material represented by 188 German and 28 Romanian genotypes [31]. The screening of genotypes with SSR markers connected to essential phenotypic traits, such as resistance to *V. pirina* [70] and *E. amylovora* [106], supplemented the previous progress made on pear regarding the evaluation of the pear gene banks or genetic factors that underlie pear responsiveness to the main biotic stresses [31,53,107,108,109,110,111]. However, because the usual selection of microsatellite loci recommends that there be at least four alleles for a microsatellite to be effective in assessing genetic diversity [112], this criterion was met in this case. The average observed heterozygosity was lower than the expected heterozygosity (Ho < He). Heterozygosity did not indicate a high level of genetic diversity among the 80 pear genotypes, even though they included a wide range of cultivars, primarily European, but also some Asian and *Pyrus* species. In addition, in the ‘species’ group, two genotypes that are actually interspecific hybrids (×*Pyronia veitkii* and ×*Sorbopyrus*) were included, which could amplify the genetic diversity. According to Postman [113], the large-fruited ×*Sorbopyrus* is a triploid selection developed in the early 1800s from a cross between *Sorbus* and *Pyrus*, and ×*Pyronia veitchii* resulted from hybridization between *Pyrus* and *Cydonia* in the early 1900s. Both the phenotypic dendrogram for the responses of the genotypes to diseases and psyllids and the molecular one highlighted their diversity by placing ×*Pyronia veitkii* and ×*Sorbopyrus* in different clusters. Regarding molecular markers, it is worth mentioning that they provide more precision in analyzing genetic diversity in germplasm collections and identifying homonymy and synonymy occurrences (and avoiding errors) [31,105,114]. In addition, one of the most important goals in reducing management costs with accessions is the avoidance of redundancy [115,116,117].

Finally, the methodologies for evaluating the frequency, severity, and degree of attack allowed for a reliable distinction of the genotypes’ responses to the studied stressors. Such studies can contribute to efforts to develop a visual rating that can distinguish disease symptoms and phenotypic differentiation of genotypes with acceptable sensitivity and safety. Visual assessment of disease and pest severity is an important technique for selection and breeding programs [118], especially for screening large gene pools, such as germplasm collections and hybrid populations. Image analysis investigations could assist in enhancing the reliability and safety of phenotypic evaluations in quantitative genetic studies for disease and pest resistance, avoiding the underestimation or overestimation of genetic factors [118]. Approaches for estimating disease severity in genetic investigations should ideally be simple, rapid, flexible, quantitative, sensitive, accurate, and repeatable [64,65,118,119]. The combination of phenotypic and molecular evaluation is helpful for obtaining relevant information in the selection of parental forms for new hybridization works and the development of new varieties with adequate pathogen and pest responses. Thus, in pear breeding programs, phenotypic evaluation of germplasm resources, identification of QTLs, and use of molecular markers have been prioritized for characterizing the genetic basis of pear resistance to the most significant pathogens and pests [54,111,120]. As a result, considerable breakthroughs in pear genetics have been made in recent decades, in addition to the creation of valuable germplasm collections [54]. Identifying the right resources will provide opportunities to make considerable progress in the development of new varieties that will help increase fruit production and quality while maintaining the environment and public health.

## 4. Materials and Methods

### 4.1. Description of the Study Site and Biological Material

The study of pear genotypes was carried out at the Horticultural Research Station (HRS) belonging to the University of Agricultural Sciences and Veterinary Medicine of Cluj-Napoca. The city of Cluj-Napoca is in Northwest Romania, where the average annual temperature is 8.2 °C and the sum of the average annual precipitation is 560 mm. The plantation with the pear genotypes is at an altitude of approximately 400 m, on degraded chernozem soil, with favorable soil and general conditions specific to the Someș Mic Valley Corridor area [121]. The land is on a slight slope, with an inclination of 8–10 degrees, with western exposure. 

All pear genotypes were grafted on the same type of rootstock (*Pyrus communis* seedings, called ‘franc’), and the planting was performed with distances between rows of 4 m and between trees in a row of 2 m, resulting in a density of 833 trees/ha. A slender spindle planting system with minimal pruning at planting was used, so that the trees would form a crown as natural as possible, with permanent scaffold branches and slight renewal pruning. The experimental pear plantation was established in 1992, comprising 365 genotypes, of which 80 were included in the current study. No tree maintenance or pruning was carried out, and phytosanitary treatments were reduced to a minimum of 3–4 treatments with specific fungicides and insecticides per year.

To analyze the responses of the genotypes to the main biotic stress factors, three distinct groups were formed, keeping the identification codes from the germplasm collection. The codes with the name of each genotype are presented in Table 1 (codes between 1 and 17 represented by 13 *Pyrus* species), Table 2 (codes between 21 and 120 represented by 17 Romanian varieties, most of them new creations), and Table 3 (codes between 122 and 296 represented by 50 non-Romanian varieties, from the international assortment).

### 4.2. Assessment of Diseases and Pests

The most prevalent diseases and pests were evaluated in the field under conditions of natural infection or infestation. The diseases included fire blight (*Erwinia amylovora*), pear scab (*Venturia pyrina*), as well as septoria (*Septoria pyricola*). In addition to pathogens, psylla species (*Psylla* sp. or *Cacopsylla* sp.) were among the pests that were most reported, and they were also examined because of the significant damage they had previously caused in the HRS’s experimental fields and neighbouring areas [37,41,42]. The assessment of disease and insect attack was carried out over four years, following the standard methodology recommended by each stressor. For this purpose, the frequency (*F*%) and intensity (*I*%) of attack were determined, and then, based on these results, the degree of attack was calculated (*DA*%) [59].

The frequency (*F*%) of attack was assessed as the relative value of the number (*n*) of plants or organs of the plant attacked by a phytopathogenic agent (fungus or bacterium) or pest (psylla) reported for the number (*N*) of plants or organs observed. The frequency value was obtained by direct observation of a number of plants or organs. The formula used was:

(1) $$F\%=\frac{n}{N}\times 100$$

The intensity (*I*%) of the attack is the coverage or spread of the attack recorded, representing the affected surface against the total observed area. It is calculated with the formula:

(2) $$I\%=\frac{\Sigma (i\times f)}{n}$$

where *i* is the class with respect to the note of attack intensity (proportion of affected organs or attacked area percent); *f* is the number of attack cases/each note; and n is the total number of attack cases. Scale or scoring classes were used to retrieve attack intensity. The classes were assigned as corresponding to certain percentage intervals of attack intensity (proportion of affected organs) with respect to notes: 0 = 0%, 1 = 1–3%, 2 = 4–10%, 3 = 11–25%, 4 = 26–50%, 5 = 51–75%, 6 = 76–100% [48,59].

**Figure 6:**
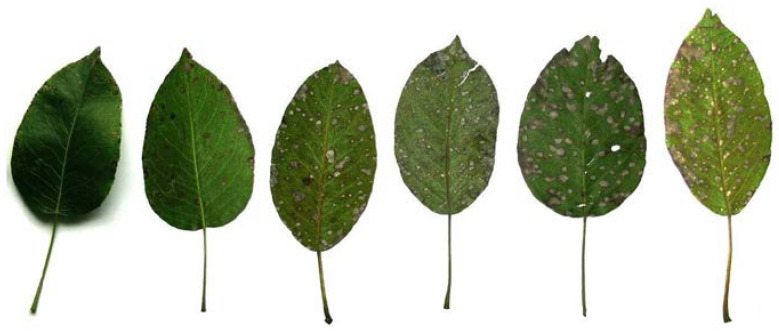
The progressive system of scale classes or grading correspondingly assigned to percentage intervals of attack intensity. For *Septoria pyricola*, the order represents the ascending grades related to the proportion of leaf damage (1 = 1–3%, 2 = 4–10%, 3 = 11–25%, 4 = 26–50%, 5 = 51–75%, 6 = 76–100%) in a system that allows a quick visual analysis for a large number of data.

The evaluations for pear scab and septoria attack were performed on leaves (a minimum of 100 leaves analyzed from two main branches and for each tree), on shoots for fire blight (all shoots with symptoms on each tree), and for the densities of eggs and nymph populations on leaves for psylla (a minimum of 100 leaves analyzed from two main branches and for each tree). For the attack intensity, the scale or scoring classes were established in accordance with the percentage ranges of the intensity of attack (with respect to the proportion of affected organs), depending on the particularities of each pathogen (Figure 6) or psyllids. 

Figure 6 shows examples of septoria or pear leaf spot (syn. ashy leaf spot of pears and leaf fleck of pears) caused by the fungus *Mycosphaerella sentina* (Fr.:Fr.) Schrot., syn. *M. pyri* (Auersw.) Boerema (anamorph: *Septoria pyricola* (Desmaz.) Desmaz.).

Since, during the study years, fire blight attack was not extremely strong and did not advance towards the branches and tree trunks, the assessment procedures proposed by Van der Zwet et al. [122] were not applied. The evaluation of the ‘susceptibility’ or ‘resistance’ of the trees to fire blight (from ‘highly resistant’ to ‘very susceptible’ classes) was reported in a reverse system to that of van der Zwet [36,93,122], considering that those trees with low scores for degree of attack (*DA*%) have better response to the disease. The formula used for degree of attack (*DA*%) was:

(3) $$DA\%=\frac{F\%\times I\%}{100}$$

Depending on the degree of attack (*DA*%) values, the following estimation of the genotypes’ reactions to biotic stressors was arbitrarily considered: 0 = no attack, 0.1–1.0 = very low attack, 1.1–5.0 = low attack, 5.1–10.0 = medium attack, 10.1–25.0 = strong attack, 25.1–50.0 = very strong attack, >50.0 = extremely strong attack (eventually drying or plant death, i.e., in the case of *Erwinia amylovora* or psylla).

### 4.3. Genetic Diversity Analysis

The genomic DNA of 80 genotypes from various species and cultivars in the collection assessed was extracted from roughly 100 mg of young leaf tissue following the SILEX protocol [123]. DNA quality and integrity were checked using NanoDrop ND–1000 spectrophotometer (Nanodrop Technologies, Wilmington, DE, USA) ratios (260/280 and 260/230) and visually on 1.0% agarose gel electrophoresis. A total of nine highly polymorphic SSRs markers, recommended by the European Cooperative Program for Plant Genetic Resources (ECPGR) [105], were screened to assess the genetic diversity of the 80 genotypes. The SSRs were PCR multiplexed, avoiding allelic overlap, and the forward primers were M13–tailed at the 5′ end with FAM or HEX fluorophores. PCR reactions were performed in a total volume of 10 uL with 1.0 uL of DNA at 20 ng/uL, 0.5 μL MgCl_2_ at 50 mM, 0.2 μL dNTPs at 10 mM, 0.2 μL forward primer at 10 mM, 0.05 μL M13–fluorescent labelled forward primer at 10 mM, 0.25 μL reverse primer at 10 mM, 0.20 μL 5 PRIME HotMaster^®^ Taq DNA Polymerase (Quantabio, Beverly, MA, USA) at 5 U/μL, 1.0 μL DNA polymerase buffer 10X, and 7.6 μL of dH_2_O, following the procedure of an initial step at 94 °C for 2 min; 30 cycles of 94 °C for 30 s, 55 °C for 30 s, and 72 °C for 30 s; and a final 10 min extension at 72 °C. PCR products were separated on an automated DNA sequencer (ABI PRISM 3100–Avant (Applied Biosystems, Waltham, MA, USA)) and analyzed with GeneScan and Genotyper software (Applied Biosystems). Genetic diversity statistics of the number of polymorphic alleles (Na), the number of genotypes (NG), the observed heterozygosity (Ho), the expected heterozygosity (He), and the polymorphic information content (PIC) for each SSR locus were calculated using PowerMaker software [124]. Using GenAlEx 6.5 software, principal coordinate analysis (PCoA) was carried out to display graphically the genetic relationships among individuals. The function aboot from the R package poppr version 2.8.1 was used to create a UPGMA dendrogram with 1000 bootstrap randomization.

### 4.4. Statistical Analysis 

Registered data recorded for the degree of attack of the pear genotypes were processed as average values and presented in the synthesis tables together with the standard errors of the means (SEMs). One-way ANOVA was applied to analyze whether the differences between genotypes in each of the three groups (species, Romanian cultivars, and non-Romanian cultivars) were significant. Before performing the ANOVA, percentage data were adjusted using the arcsine transformation. If the null hypothesis was rejected, Duncan’s multiple range test (Duncan’s MRT, *p* < 0.05) was used as the post hoc test for the analysis of differences. The data were subjected to multivariate statistical analysis performed using Past software [125]. Hierarchical clustering, paired groups (UPGMA—unweighted pair group method with arithmetic mean), and Euclidean similarity indexes were computed for all pear genotypes and analyzed attributes.

## Figures and Tables

**Table 1:** Responses to the principal diseases and pests of 13 pear species (as degree of attack, *DA*%) ^1^.

Code	Species	*Venturia* *pyrina*	*Septoria* *pyricola*	*Erwinia* *amylovora*	*Psylla* sp.
1	×*Pyronia veitkii*	9.4 ± 0.6 ^a^	2.8 ± 0.2 ^a^	0.0 ± 0.0 ^e^	1.7 ± 0.4 ^b^
2	*Pyrus betulaefolia*	3.8 ± 0.6 ^d^	2.2 ± 0.5 ^a,b^	20.6 ± 3.6 ^b,c^	0.3 ± 0.0 ^d^
4	*Pyrus caucasica*	6.7 ± 0.7 ^b^	2.6 ± 0.9 ^a^	0.0 ± 0.0 ^e^	1.4 ± 0.4 ^b,c^
5	*Pyrus pyraster*	5.5 ± 0.7 ^b,c^	2.0 ± 0.4 ^a–c^	0.0 ± 0.0 ^e^	0.1 ± 0.0 ^d^
6	*Pyrus communis*	4.8 ± 0.8 ^c,d^	3.4 ± 0.6 ^a^	0.4 ± 0.1 ^e^	3.9 ± 1.2 ^a^
7	*Pyrus cordata*	1.5 ± 0.4 ^f,g^	2.4 ± 0.8 ^a,b^	19.2 ± 2.7 ^b,c^	0.5 ± 0.1 ^c,d^
9	*Pyrus eleagrifolia*	0.5 ± 0.2 ^g^	1.6 ± 0.3 ^a–c^	24.1 ± 1.6 ^b^	0.4 ± 0.1 ^c,d^
12	*Pyrus lindlezi*	0.2 ± 0.1 ^g^	2.1 ± 0.2 ^a,b^	0.0 ± 0.0 ^e^	0.0 ± 0.0 ^d^
13	*Pyrus longipes*	0.2 ± 0.1 ^g^	1.5 ± 0.3 ^a–c^	7.3 ± 0.5 ^d^	0.3 ± 0.0 ^d^
14	*Pyrus malifolia*	3.6 ± 0.8 ^d,e^	3.4 ± 1.5 ^a^	0.0 ± 0.0 ^e^	0.2 ± 0.0 ^d^
15	*Pyrus nivalis*	0.4 ± 0.3 ^g^	0.4 ± 0.1 ^b,c^	32.4 ± 3.4 ^a^	0.1 ± 0.0 ^d^
16	*Pyrus persica*	0.0 ± 0.0 ^g^	0.0 ± 0.0 ^c^	0.0 ± 0.0 ^e^	0.0 ± 0.0 ^d^
17	×*Sorbopyrus*	2.3 ± 0.4 ^e,f^	0.5 ± 0.2 ^b,c^	17.3 ± 1.6 ^c^	0.0 ± 0.0 ^d^
	Average value	3.0	1.9	9.3	0.7

^1^ Different letters between genotypes in each column indicate statistically significant differences for the investigated feature at a significance level of *p* < 0.05 (Duncan’s test).

**Table 2:** Responses to the principal diseases and pests of 17 Romanian pear cultivars (as degree of attack, *DA*%) ^1^.

Code	Cultivar	*Venturia* *pyrina*	*Septoria* *pyricola*	*Erwinia* *amylovora*	*Psylla* sp.
21	Argessis	4.8 ± 0.5 ^a–c^	0.8 ± 0.2 ^e–h^	19.3 ± 3.1 ^b^	2.6 ± 0.5 ^c–e^
28	Cântări	5.9 ± 1.0 ^a^	1.9 ± 0.3 ^a–d^	20.7 ± 4.7 ^b^	1.6 ± 0.3 ^d,e^
34	Cu miez roşu	2.0 ± 0.3 ^f,g^	2.0 ± 0.2 ^a–c^	15.1 ± 3.3 ^b,c^	1.9 ± 0.3 ^d,e^
88	Republica	5.5 ± 0.9 ^a^	0.3 ± 0.2 ^h^	33.7 ± 4.8 ^a^	1.9 ± 0.2 ^d,e^
106	Zaharoasă de vară	3.2 ± 0.7 ^d–f^	1.6 ± 0.5 ^b–e^	0.0 ± 0.0 ^f^	2.5 ± 0.9 ^c–e^
107	Adria	2.7 ± 0.4 ^d–g^	1.2 ± 0.2 ^d–g^	0.0 ± 0.0 ^f^	1.0 ± 0.0 ^e^
109	Doina	3.5 ± 0.3 ^c–e^	2.1 ± 0.2 ^ab^	14.8 ± 2.7 ^b–d^	4.9 ± 1.1 ^a,b^
110	Haydeea	0.1 ± 0.0 ^h^	0.8 ± 0.1 ^e–h^	0.0 ± 0.0 ^f^	0.4 ± 0.1 ^e^
111	Ina Estival	1.7 ± 0.4 ^g^	1.1 ± 0.2 ^e–g^	0.0 ± 0.0 ^f^	2.1 ± 0.5 ^d,e^
112	Jubileu 50	2.5 ± 0.6 ^e–g^	0.6 ± 0.1 ^g,h^	7.6 ± 1.1 ^e^	2.1 ± 0.1 ^d,e^
114	Meda	4.0 ± 0.3 ^b–d^	2.0 ± 0.2 ^a–c^	0.0 ± 0.0 ^f^	3.3 ± 0.9 ^b–d^
115	Milenium	2.8 ± 0.4 ^d–g^	2.5 ± 0.4 ^a^	8.1 ± 1.5 ^d,e^	4.1 ± 1.1 ^a–c^
116	Napoca	1.7 ± 0.4 ^g^	1.4 ± 0.3 ^c–f^	0.0 ± 0.0 ^f^	2.6 ± 0.8 ^c–e^
117	Primadona	3.7 ± 0.6 ^c–e^	0.2 ± 0.1 ^h^	6.6 ± 1.4 ^e^	2.1 ± 0.1 ^d,e^
118	Roșioară de Cluj	4.8 ± 0.6 ^a–c^	2.1 ± 0.1 ^a,b^	10.2 ± 2.0 ^c–e^	5.1 ± 1.1 ^a^
119	Transilvania	2.0 ± 0.3 ^f,g^	1.9 ± 0.2 ^a–d^	8.9 ± 0.8 ^c–e^	2.3 ± 0.6 ^c–e^
120	Virgiliu Hibernal	5.3 ± 0.7 ^a,b^	2.0 ± 0.4 ^a–c^	9.1 ± 2.6 ^c–e^	5.0 ± 0.6 ^a,b^
	Average value	3.3	1.4	9.1	2.7

^1^ Different letters between genotypes in each column indicate statistically significant differences for the investigated feature at a significance level of *p* < 0.05 (Duncan’s test).

**Table 3:** Response to the principal diseases and pests of 50 non-Romanian (international) pear cultivars (as degree of attack, *DA*%) ^1^.

Code	Cultivar	*Venturia* *pyrina*	*Septoria* *pyricola*	*Erwinia* *amylovora*	*Psylla* sp.
122	Abate Fetel	6.7 ± 2.2 ^d–g^	0.4 ± 0.1 ^e–g^	35.3 ± 5.8 ^a–d^	1.3 ± 0.3 ^i–m^
127	Arabitka	5.3 ± 1.2 ^e–h^	2.0 ± 0.6 ^c,d^	4.3 ± 0.9 ^k–n^	3.7 ± 0.9 ^f–k^
136	Bergamotte Esperen	4.0 ± 0.6 ^g–i^	1.7 ± 0.3 ^c–e^	20.0 ± 6.0 ^c–k^	4.0 ± 1.0 ^f–j^
137	Beurré Amanlis	3.0 ± 0.6 ^g–i^	1.3 ± 0.3 ^d–g^	0.0 ± 0.0 ^n^	8.3 ± 1.5 ^c,d^
138	Beurré Bachelier	2.4 ± 0.8 ^h,i^	0.5 ± 0.1 ^e–g^	0.0 ± 0.0 ^n^	7.7 ± 1.5 ^c–e^
139	Beurré Bosc	3.3 ± 0.9 ^g–i^	0.9 ± 0.3 ^d–g^	1.8 ± 0.4 ^m,n^	4.0 ± 0.6 ^f–j^
142	Beurré Diel	3.0 ± 0.6 ^g–i^	1.5 ± 0.3 ^d–f^	21.7 ± 5.8 ^c–j^	3.0 ± 0.6 ^f–m^
146	Beurré du Luçon	1.6 ± 0.3 ^h,i^	0.5 ± 0.1 ^e–g^	0.0 ± 0.0 ^n^	1.0 ± 0.7 ^i–m^
152	Blanquet Precoce	1.5 ± 0.3 ^h,i^	0.8 ± 0.2 ^d–g^	0.0 ± 0.0 ^n^	4.7 ± 0.9 ^e–h^
154	Bonne Louise d’Avranches	1.5 ± 0.3 ^h,i^	1.1 ± 0.3 ^d–g^	36.7 ± 6.9 ^a–c^	8.7 ± 1.2 ^c,d^
155	Bristol Cross	1.6 ± 0.4 ^h,i^	1.5 ± 0.3 ^d–f^	0.0 ± 0.0 ^n^	2.0 ± 0.6 ^g–m^
156	Bunte Julibirne	1.1 ± 0.1 ^j^	0.6 ± 0.2 ^e–g^	40.7 ± 9.3 ^a^	2.1 ± 0.6 ^g–m^
157	Butirra Precoce Morettini	0.9 ± 0.1 ^j^	1.2 ± 0.1 ^d–g^	40.7 ± 6.8 ^a,b^	11.7 ± 4.7 ^b^
162	Chang Pa Li	1.3 ± 0.2 ^h,i^	0.6 ± 0.2 ^e–g^	39.7 ± 11.3 ^a,b^	0.8 ± 0.2 ^i–m^
167	Conference	8.0 ± 1.7 ^c–e^	4.3 ± 1.2 ^b^	0.0 ± 0.0 ^n^	4.2 ± 1.2 ^f–i^
168	Conseiller de la Cour	3.1 ± 0.6 ^g–i^	1.5 ± 0.3 ^d–f^	0.0 ± 0.0 ^n^	4.3 ± 0.7 ^f–i^
169	Curé	1.0 ± 0.2 ^j^	0.6 ± 0.2 ^e–g^	2.0 ± 0.6 ^m,n^	0.1 ± 0.0 ^l,m^
173	Beurré Hardy	1.0 ± 0.1 ^j^	0.3 ± 0.1 ^f,g^	3.0 ± 0.6 ^l–n^	2.0 ± 0.6 ^g–m^
179	Dr. Jules Guyot	2.8 ± 0.6 ^h,i^	0.7 ± 0.2 ^d–g^	25.7 ± 8.8 ^b–f^	1.7 ± 0.3 ^h–m^
187	Beurré Giffard	6.7 ± 0.9 ^d–g^	0.5 ± 0.1 ^e–g^	7.3 ± 2.3 ^i–n^	2.0 ± 0.6 ^g–m^
188	Er Shi Shinge	0.0 ± 0.0 ^k^	0.5 ± 0.1 ^e–g^	1.0 ± 0.5 ^n^	0.2 ± 0.0 ^l,m^
189	Er Jang Li	4.2 ± 0.4 ^f–i^	0.4 ± 0.1 ^e–g^	21.7 ± 6.2 ^c–j^	4.7 ± 0.3 ^e–h^
194	Fondante des Bois	10.0 ± 1.5 ^c^	1.3 ± 0.4 ^d–g^	25.7 ± 9.0 ^b–f^	3.3 ± 0.3 ^f–l^
197	General Leclerc	5.3 ± 1.2 ^e–h^	0.4 ± 0.1 ^e–g^	12.3 ± 4.4 ^f–n^	1.3 ± 0.3 ^i–m^
198	General Osmanwill	8.0 ± 0.6 ^c–e^	0.3 ± 0.1 ^f,g^	0.0 ± 0.0 ^n^	0.3 ± 0.1 ^l,m^
202	Grand Champion	10.7 ± 0.9 ^c^	4.3 ± 1.2 ^b^	0.0 ± 0.0 ^n^	4.0 ± 0.6 ^f–j^
209	Imperial	1.2 ± 0.1 ^j^	0.3 ± 0.1 ^f,g^	2.3 ± 0.9 ^m,n^	7.7 ± 1.8 ^c–e^
213	Juliusi Selimesi	1.4 ± 0.3 ^h,i^	0.4 ± 0.1 ^e–g^	3.7 ± 0.9 ^l–n^	1.7 ± 0.3 ^h–m^
221	Kostliche Von Germen	15.0 ± 1.5 ^b^	1.1 ± 0.3 ^d–g^	6.3 ± 3.0 ^j–n^	4.7 ± 1.5 ^e–h^
222	Kristalli	0.0 ± 0.0 ^k^	7.0 ± 1.2 ^a^	22.0 ± 4.9 ^c–j^	14.7 ± 3.2 ^a^
224	Laurence	3.0 ± 0.6 ^g–i^	1.2 ± 0.2 ^d–g^	0.0 ± 0.0 ^n^	3.3 ± 0.9 ^f–l^
225	Laxton Superb	20.3 ± 1.2 ^a^	0.5 ± 0.2 ^e–g^	4.3 ± 1.2 ^k–n^	0.1 ± 0.0 ^l,m^
229	Lincoln	8.3 ± 1.8 ^c–e^	1.2 ± 0.4 ^d–g^	17.7 ± 3.3 ^e–m^	2.3 ± 0.9 ^g–m^
232	Madame Ballet	7.7 ± 2.0 ^c–f^	3.3 ± 0.9 ^b,c^	9.0 ± 1.2 ^h–n^	0.2 ± 0.1 ^l,m^
233	Magness	4.0 ± 1.5 ^g–i^	0.3 ± 0.1 ^f,g^	0.0 ± 0.0 ^n^	0.3 ± 0.1 ^l,m^
240	Moonglow	18.3 ± 3.7 ^a^	0.4 ± 0.1 ^e–g^	32.0 ± 5.8 ^a–e^	0.7 ± 0.1 ^k–m^
246	Noiabriscaia	10.3 ± 1.9 ^c^	0.8 ± 0.1 ^d–g^	24.3 ± 6.9 ^c–h^	9.7 ± 1.2 ^b,c^
248	Okusankichi	0.0 ± 0.0 ^k^	0.6 ± 0.1 ^e–g^	25.7 ± 10.1 ^b–f^	0.1 ± 0.0 ^l,m^
250	Olivier de Serres	0.0 ± 0.0 ^k^	0.5 ± 0.2 ^e–g^	24.7 ± 8.6 ^c–g^	0.0 ± 0.0 ^m^
258	Pierre Corneille	2.3 ± 0.3 ^h,i^	0.2 ± 0.1 ^e–g^	22.7 ± 5.8 ^c–i^	1.0 ± 0.5 ^i–m^
259	Pitmaston Duchess	2.0 ± 0.6 ^h,i^	0.7 ± 0.1 ^d–g^	18.7 ± 2.3 ^e–l^	1.7 ± 0.3 ^h–m^
260	Plovdivka Parva	0.3 ± 0.3 ^j^	0.6 ± 0.1 ^e–g^	4.0 ± 1.5 ^l–n^	1.3 ± 0.3 ^i–m^
263	Précoce Trottier	0.0 ± 0.0 ^k^	0.5 ± 0.2 ^e–g^	0.0 ± 0.0 ^n^	0.1 ± 0.0 ^l,m^
272	Seigneur Esperen	2.0 ± 0.6 ^h,i^	0.5 ± 0.1 ^e–g^	0.0 ± 0.0 ^n^	1.3 ± 0.3 ^i–m^
286	Triomphe de Jodoigne	14.0 ± 3.5 ^b^	0.6 ± 0.1 ^e–g^	9.0 ± 1.2 ^h–n^	0.2 ± 0.1 ^l,m^
288	Triomphe de Vienne	2.0 ± 0.6 ^h,i^	0.6 ± 0.2 ^e–g^	18.3 ± 3.7 ^e–l^	2.3 ± 0.9 ^g–m^
289	Van Mons	9.3 ± 0.9 ^c,d^	2.7 ± 1.2 ^c^	10.0 ± 2.1 ^g–n^	1.7 ± 0.3 ^h–m^
293	Williams	0.7 ± 0.2 ^j^	0.2 ± 0.1 ^h^	0.0 ± 0.0 ^n^	5.0 ± 0.6 ^e–g^
294	Williams Bovey	0.9 ± 0.1 ^j^	0.1 ± 0.1 ^h^	0.0 ± 0.0 ^n^	4.0 ±0.6 ^f–j^
296	Williams Red	1.0 ± 0.2 ^j^	1.1 ± 0.1 ^d–g^	35.0 ± 6.1 ^a–d^	6.0 ± 0.6 ^d–f^
	Average value	4.4	1.1	12.6	3.2

^1^ Different letters between genotypes in each column indicate statistically significant differences for the investigated feature at a significance level of *p* < 0.05 (Duncan’s test).

**Table 4:** Genetic diversity parameters ^1^ of 80 pear genotypes represented by 13 *Pyrus* species, 17 Romanian cultivars, and 50 non-Romanian/international cultivars, assessed by molecular marker analysis, using nine SSR markers.

SSR Marker	Major Allele Frequency	NG	No. Obs.	Na	Ava	Ho	He	PIC
01D08	0.322	24	59	13	0.738	0.729	0.796	0.769
04E03A	0.620	6	79	4	0.988	0.608	0.543	0.485
04E03B	0.297	18	59	12	0.738	0.237	0.823	0.803
03D12	0.278	32	72	18	0.900	0.611	0.824	0.805
01D09	0.232	36	56	21	0.700	0.607	0.880	0.870
EMPC117	0.350	25	70	16	0.875	0.443	0.758	0.726
01F07A	0.257	34	72	20	0.900	0.764	0.864	0.851
03G07	0.544	24	68	14	0.850	0.471	0.669	0.647
05C06A	0.571	19	77	13	0.963	0.247	0.642	0.621

^1^ NG—number of genotypes; No. Obs.—number of observations; Na—number of alleles per locus; Ava—availability; Ho—observed heterozygosity; He—expected heterozygosity; PIC—polymorphism information content.

## Data Availability

Not applicable.
